# Sepsis in HIV patients admitted to the ICU

**DOI:** 10.1186/cc12451

**Published:** 2013-03-19

**Authors:** P Vidal-Cortés, P Lameiro-Flores, M Mourelo-Fariña, A Aller-Fernández, R Gómez-López, P Fernández-Ugidos, M Alves-Pérez, E Rodríguez-García

**Affiliations:** 1 CHU Ourense, Spain; 2 CHU A Coruña, Spain

## Introduction

Our objective was to analyze septic HIV patients admitted to intensive care.

## Methods

A retrospective study of HIV patients admitted to our ICU between January 2005 and December 2009. We identify patients admitted to the ICU with sepsis and analyze demographic factors, etiology, organ failure and outcome, and we compare immune status, frequency of organ failure and outcome between these patients and those admitted for other reasons. We use Student's *t *test to compare quantitative variables, and the chi-square test for qualitative data.

## Results

A total of 104 HIV patients were admitted to our ICU during the study period, 62 with sepsis (71% men, mean age: 40.59 ± 8.12). Of sepsis patients, 56.5% were admitted from the ER and 38.7% from a medical ward; 66.1% had history of intravenous drugs use, other comorbidities: COPD (9.7%), cirrhosis (8.1%), solid or hematologic malignancy (12.9%); 8.1% patients were unaware of their condition (2.4% in non-infected patients, *P *= 0.223) and 40.3% were under HAART (64.3% in patients admitted without infection, *P *= 0.016). Mean CD4 count at admission: 219.62 ± 353.93 cells/mm^3 ^(vs. 370.22 ± 362.56, *P *= 0.048). Mean viral load: 4.57 ± 3.25 log (vs. 2.257 ± 1.96, *P *= 0.001). Of sepsis patients, 62.1% were on their CD4 nadir (vs. 34.5% in nonseptic patients, *P *= 0.009). Mean albumin levels were 2.3 ± 0.53 g/ dl (vs. 2.92 ± 0.94, *P *0.001). APACHE II at admission was 21.98 ± 7.97 (vs. 18.15 ± 8.47, *P *= 0.046). At admission, 52.8% were on severe sepsis and 44.3% on septic shock. The lung was the most frequent source of infection (65.6%) followed by CNS (16.4%), UTI (4.9%) and IE (4.9%). The most common pathogen isolated on these patients was *S. pneumoniae *(28.8%), followed by *P. jirovecii *(13.6%), toxoplasma (8.5%), *E. coli *(5.1%) and *H. influenzae *(5.1%). In total, 62.9% needed vasopressors (vs. 28.6% in non-infected patients, *P *= 0.001), 79% mechanical ventilation (vs. 42.85%, *P *0.001) and 19.4% renal replacement (9.5% in no septic patients, *P *= 0.173). Mean ICU and hospital LOS was 10.43 ± 10.52 and 34.76 ± 29.64 days in septic patients versus 6.04 ± 8.45 (*P *= 0.026) and 20.54 ± 27.93 days (*P *= 0.016). ICU mortality: 33.9% (19% in nonseptic patients, *P *= 0.098), hospital mortality: 41.9% (vs. 23.8%, *P *= 0.057).

## Conclusion

Sepsis is a common reason for admission to the ICU in HIV patients and is accompanied by high mortality. Pneumonia is the most frequent source of infection. Septic patients are less frequently under HAART and have a worse inmune status (lower CD4 count and higher viral load). Despite a higher APACHE II, and a higher need for hemodynamic and respiratory support, there is no statistically significant difference in ICU and hospital mortality between septic and nonseptic patients.

